# How to unmask septal local abnormal ventricular activities with the new LUMIPOINT^TM^ software

**DOI:** 10.1002/joa3.12344

**Published:** 2020-05-01

**Authors:** Francesco Solimene, Francesco Maddaluno, Maurizio Malacrida, Giuseppe Stabile

**Affiliations:** ^1^ Clinica Montevergine Mercogliano Avellino Italy; ^2^ Boston Scientific Italy Milan Italy

**Keywords:** high‐density mapping system, LAVA, lumipoint, RF ablation, ventricular tachycardia

## Abstract

We report the use of the new automated tool Lumipoint^TM^ for the detection of LAVA (local abnormal ventricular activities) when they are buried within the far‐field ventricular signal, especially in regions of preserved myocardial thickness, such as the left ventricular (LV) septum. The LV substrate and the tachycardia circuit during ventricular tachycardias of a 60‐year‐old man with dilated cardiomyopathy were mapped using an ultra‐high‐density mapping system and then the Lumipoint^TM^, analyzing the EGMs of interest, identified the LAVA in the inferoseptal region. This algorithm may be helpful to quickly target the septal substrate avoiding misleading interpretation.

## Graphical abstract

The Lumipoint tool allows electrophysiologists to quickly identify and target the septal substrate analyzing thousands of electrograms and illuminating signals of interest on the map.

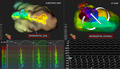

**Abbreviations**
EGM: electrogram
ICD: implantable defibrillator
LAVA: local abnormal ventricular activities
LV: left ventricle
RAO: right anterior oblique view
RF: radiofrequency
RV: right ventricle
VT: ventricular tachycardias

## 1 INTRODUCTION

Local abnormal ventricular activities (LAVA) are defined as sharp high‐frequency ventricular potentials, distinct from far‐field ventricular electrograms (EGM), which can be detected during sinus or paced rhythms.^1^ The chance of detecting LAVA increases when EGM onset is late in relation to the QRS complex. Indeed, in early activated regions, such as the left ventricular septum during sinus rhythm or right ventricular (RV) pacing, LAVA may be buried within the far‐field ventricular potential, displaying a timing similar to the QRS onset.^2^ When LAVA detection overlaps with the far‐field ventricular signal, several time‐consuming maneuvers, such as changing activation wavefronts^3^ or ventricular extra stimulus,^1^ have been introduced in order to identify the septal arrhythmogenic substrate. Here, we describe the use of a new automated tool that can help the physician to quickly identify and target the septal substrate.

## 2 CASE DESCRIPTION

**FIGURE 1:**
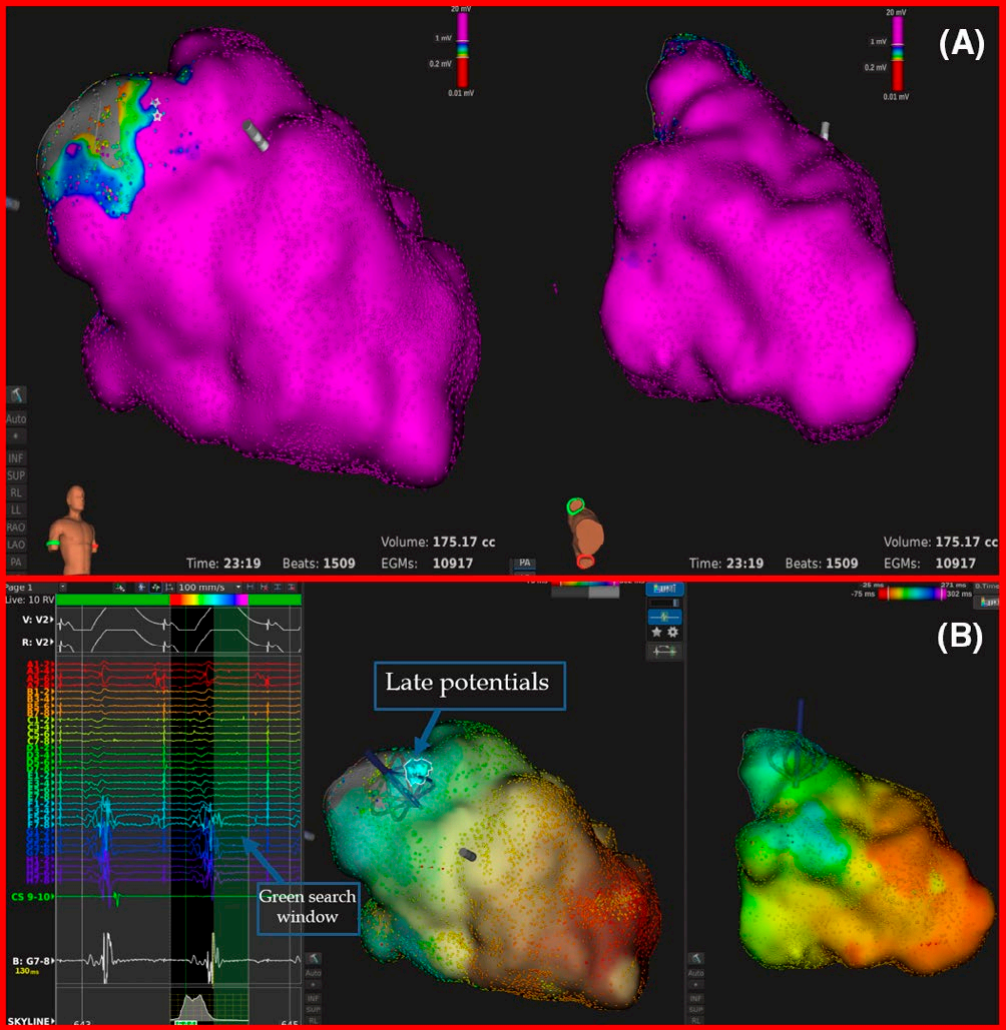
A, Voltage map acquired during RV pacing shows signal amplitude greater than 1 mV (purple color) in every region of the LV, the only exception being the sub‐aortic region (gray zone). Left panel shows a right anterior oblique (RAO) view, whereas right panel shows a RAO view from below, enabling the inferoseptal wall to be visualized. B, Activation map acquired during RV pacing. Left panel shows a RAO view, whereas right panel shows a RAO view from below, the inferoseptal wall to be visualized. The Lumipoint^TM^ simple activation search tool allows the operator to open a green search window inside the mapping window so that the software will illuminate every EGM on the map that has a marked deflection falling within that search window, irrespective of whether or not that deflection has been chosen as the EGM annotation on the activation map.^4^ By positioning the green search window after the QRS offset, a small region of late potentials was illuminated in the anterior sub‐aortic region

**FIGURE 2:**
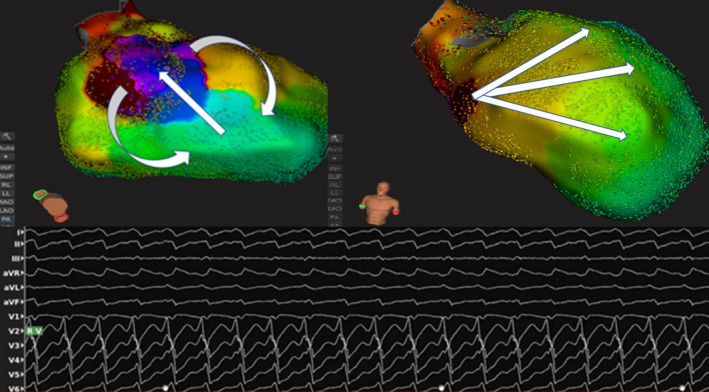
This picture represents a frame of the propagation map during the clinical VT. Left panel shows a lifted RAO view where the critical isthmus can be seen. Right panel is an AP projection, where it can be seen that most of the LV is centrifugally activated from the septum

**FIGURE 3:**
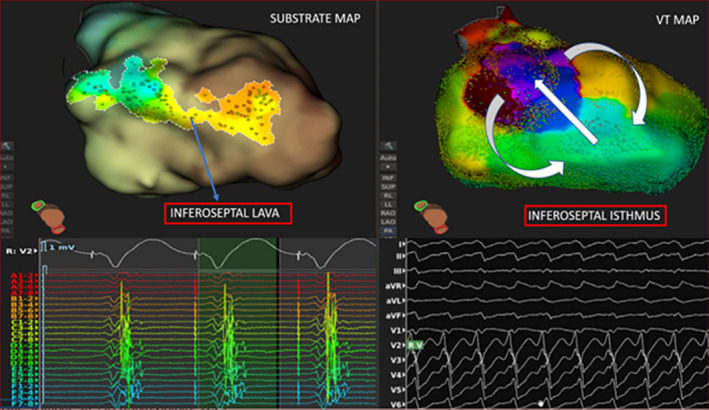
Left panel. The Lumipoint^TM^ green search window is extended into the QRS and the software is asked to illuminate fragmented potentials. A quite extensive substrate is highlighted along the inferoseptal wall. A quick look at the signals of the Orion splines in contact with that region (splines C, D, and E) shows the multiphasic nature of the EGM: the first component is a low‐amplitude far‐field signal from the RV, the second component is a high‐voltage far‐field signal from the LV septum, and the remaining part of the signal is the abnormal activity, which is partly fused with the previous component. The spot identified by the arrow in the substrate map is also the exact point where clinical VT was interrupted. Right panel: activation map during the clinical VT showing the presence of the critical isthmus at the inferoseptal wall. Both projections are a RAO view from below, enabling clear visualization of the LV infero‐septum

A 60‐year‐old man with dilated cardiomyopathy was referred to our laboratory for recurrent episodes of ventricular tachycardias (VTs), which were usually terminated by high‐energy shocks from his dual‐chamber implantable defibrillator (ICD). We mapped both the left ventricular (LV) substrate and the tachycardia circuit during VT using the Orion multipolar basket catheter and the Rhythmia mapping system (Boston Scientific). The substrate map was acquired during RV pacing (Figure 1A). At this point, we used the new Lumipoint^TM^ algorithm to rapidly look for EGMs of interest^4^ (Figure 1B). Although both the voltage map and the presence of late activity in the activation map pointed to the sub‐aortic region as the arrhythmogenic substrate, activation mapping of the clinical VT revealed that the critical isthmus was entirely confined to the inferoseptal region (Figure 1B right panel and supplementary video and Figure 2). On creating radiofrequency (RF) lesions in the inferoseptal region with displayed mid‐diastolic EGMs, VT was easily interrupted. At this point, we returned to the substrate map and better analyzed the signals detected in the inferoseptal region where early activated LAVA could be seen in addition to high‐voltage far‐field signals. By extending the Lumipoint^TM^ search window inside the QRS and asking the software to illuminate fragmented potentials, we saw that LAVA spanned the whole inferoseptal wall from the base to the apex (Figure 3). After homogenization of this area (RF energy at 40 Watts for at least 30 seconds was delivered at each point of the inferoseptal wall highlighted by the fragmented tool of the Lumipoint^TM^ software until the low‐amplitude LAVA disappeared), VT was no longer inducible. After a follow‐up of 7 months, no VT recurrence was reported by the patient or detected by his ICD.

## 3 DISCUSSION

LAVA may appear at any time during or after the far‐field ventricular signal during sinus or paced rhythm, and their lateness depends on both the local conduction delay and the anatomic location of the arrhythmogenic region.^2^ In early activated regions, such as the LV septum, LAVA may appear fused within the far‐field signal generated by healthy myocardium; this means that, in clinical practice, their identification is not simple and depends mainly on the subjective judgment of the operator.^5^ To further complicate the identification of septal LAVA, far‐field voltages in areas of preserved wall thickness are quite normal, and the automated peak‐to‐peak voltage annotation of mapping systems may be only the expression of far‐field voltages that in no way indicate the presence of small‐amplitude LAVA. This inherent limitation of automated mapping systems, which preferentially annotate the far‐field voltage component in thicker areas (like the septum), is a further challenge in spotting early activated LAVA, as demonstrated by our case. Given these difficulties, some time‐consuming maneuvers have been developed in order to help the physician unmask septal LAVA. The key to confirming the presence of LAVA and to distinguishing them from far‐field potentials is to demonstrate that they are poorly coupled to the rest of the myocardium. The fractionated tool of Lumipoint^TM^ can address the physician to look at signals in a region where a fragmented activity exists. In this case, the fragmented activity was found inside the QRS complex. Normal tissue typically does not show fragmented signals but just a single rapid deflection inside the QRS; therefore, if a fragmented activity is found inside the QRS, which means that in addition to the far field from normal tissue, there are other sharp ventricular potentials and this is exactly the definition of LAVA. So, if a fragmented activity is found by the Lumipoint^TM^ software inside the QRS in a specific region, this means that the same region harbors LAVA. At this point, by looking at the LAVA signals, the electrophysiologist can differentiate the high‐amplitude far‐field from the lower‐amplitude near‐field LAVA.

When the area suspected of harboring LAVA is quite extensive, these pacing maneuvers have to be carried out throughout the region of interest, with the risk of unmasking fewer abnormal EGMs than originally thought. Another maneuver used in order to find a poorly coupled near‐field potential is pacing at a fixed rate from the catheter recording the suspected LAVA. When a long stimulus‐to‐QRS (greater than 40 ms) is detected, slow conduction away from the pacing site indicates an abnormal substrate.^5^ Once again, however, the area from which to stimulate is subjective, and critical substrates can be missed.

The nature of fractionation split and the lateness of septal LAVA are strongly influenced by the activation wavefront. However, despite the introduction of ultra‐high‐density mapping systems, it is still time‐consuming in daily practice to create multiple substrate maps (as through RV and LV pacing). The fractionation tool of the new Lumipoint^TM^ software is a new automated algorithm that is able to illuminate areas of the LV whose EGMs have multiple deflections. This tool is very effective in spotting septal LAVA, as indicated by our case, since it eliminates the subjectivity of the human eye in assessing high‐frequency signals that are buried within, or very close in activation to, far‐field signals. The software analyses thousands of EGMs in a matter of seconds, providing a clear identification of the septal region harboring signals with multiple deflections. The same analysis by the eye of the physician would be much more time‐consuming and would inevitably introduce some subjectivity. Even if the physician applies pacing maneuvers to the signals illuminated by the software, in order to confirm the nature of LAVA, the process depends on the rapid identification of a very likely septal substrate. In addition to this, the Lumipoint^TM^ software is also a reannotation tool, and it can be used to correctly annotate LAVA instead of far field.

## 4 CONCLUSIONS

In our case, the new Lumipoint^TM^ algorithm was helpful in rapidly identifying septal LAVA, which constituted the critical substrate of the clinical VT.

## CONFLICT OF INTEREST

No external funding was obtained for this project. Francesco Maddaluno and Maurizio Malacrida are Boston Scientific employees, no other conflicts of interest exist.

## Supporting information

Video S1

Video S2

Video S3

Supplementary Material

Supplementary Material

Supplementary Material
